# American Veterinary College

**Published:** 1883-04

**Authors:** 


### AMERICAN VETERINARY COLLEGE.

The Eighth Annual Commencement exercises of the American Veterinary College were held in Chickering Hall on the evening of March 1st. Mr. Samuel Marsh presided, and there were also on the platform Prof. Liautard, Dean of the Faculty, and others. Prof. Charles A. Doremus awarded the special prizes. Franklin J. Hanshew received a gold medal for passing the best general examination. Two standard veterinary works were awarded to Harris L. Alderman, and a gold medal and a set of surgical instruments were presented to Richard Kay, of California. W. R. J. Mitchell, of the Class of '84, received a silver medal for passing the best examination in his class. Samuel K. Johnson, of the gradnating class, delivered the valedictory address, and an address to the graduating class was delivered by the Rev. Henry Ward Beecher. Mr. Beecher said: "Human life may be of more importance than the life of an animal, and yet the veterinary surgeon may rank as high as him who ministers to humanity. A man need not necessarily be an ass because he cares for horses. The general structure of mammals below man was the same as the structure of human beings. Their functions were the same. In the study of the lower animal kingdom men ranked as high as those who studied the human body. Huxley, Lubbock and Darwin earned their fame largely by the study of the lower animals. If ever there was a faithful animal it was the horse. In the opalescent vision of St. John in the Apocalypse the horse was deemed worthy of being associated with the gods. Loving liberty, how readily he submits to bondage. He is ten times stronger than man, and yet how submissive to man's will! If a horse but knew his rights and his power, no man could abuse or maltreat him. His self-abnegation deserved a better fate. 'He is the servant of all and the slave of all and abused of all.' He begins life with one year as a colt, and when at last he has ceased to be useful to the peddler he has 'the only privilege of his life—the privilege of dying.' In war the horse is 'as sensitive to danger as the most nervous of men. When the trumpet calls he swallows his fear and offers his life as readily as a brave and patient man. Yet for him there is no reward, no glittering medal, no honorable mention in the Gazette, and no pension.' The draft horse was more to be admired than the racer. He was the family friend. What a debt was due him which was never repaid! 'The physician uses him from door to door, and collects his inevitable and inexorable fee, but the horse gets nothing but the privilege of going again, and often without even an 'oat-stiver.' It ought to give a man pleasure to be called to minister to the sufferings of this most human and most abused of all animals.' Then there was the cow, 'not the one that pastures at the pump, nor the distillery cow that the devil fosters, but the cow that lies under the shadowy trees in Summer and looks as sleepy as an August clergyman.' She was the best physician for children. This is an age of humanity," he said, in conclusion. "Men are sensitive to suffering as they never were before. Cruel laws are passing away, and even cruelty in slaughtering animals is discountenanced. Do not let any man look down on you because he ministers to mankind while you minister to suffering brutes. Let your names be remembered for your fidelity, your humanity and your science."

Diplomas were conferred upon the following named members of the graduating class: Harry Louis Alderman, William Henry Arrowsmith, Henry William Bath, William C. Bretherton, Eugene Berget, Lemuel C. Campbell, William Dana Critcherson, Irving S. Denslow, Christmas Evans, Julian Edward Gardner, Franklin Joseph Hanshew, Joseph R. Hodgson, Frederick Willis Huntington, Samuel K. Johnson, Franklin May Kain, Richard Kay, Arthur B. Morse, John Allebaugh Myers, W. Bertram Carnes Noyes, William Hamilton Pendry, Austin Peters, B. S., James F. Ryder.

